# Resolving social conflict among females without overt aggression

**DOI:** 10.1098/rstb.2013.0076

**Published:** 2013-12-05

**Authors:** Michael A. Cant, Andrew J. Young

**Affiliations:** Centre for Ecology and Conservation, University of Exeter, Tremough Campus, Penryn, Cornwall TR10 8BG, UK

**Keywords:** reproductive skew, fighting, threats, conflict resolution, negotiation, evolution of cooperation

## Abstract

Members of animal societies compete over resources and reproduction, but the extent to which such conflicts of interest are resolved peacefully (without recourse to costly or wasteful acts of aggression) varies widely. Here, we describe two theoretical mechanisms that can help to understand variation in the incidence of overt behavioural conflict: (i) destruction competition and (ii) the use of threats. The two mechanisms make different assumptions about the degree to which competitors are socially sensitive (responsive to real-time changes in the behaviour of their social partners). In each case, we discuss how the model assumptions relate to biological reality and highlight the genetic, ecological and informational factors that are likely to promote peaceful conflict resolution, drawing on empirical examples. We suggest that, relative to males, reproductive conflict among females may be more frequently resolved peacefully through threats of punishment, rather than overt acts of punishment, because (i) offspring are more costly to produce for females and (ii) reproduction is more difficult to conceal. The main need now is for empirical work to test whether the mechanisms described here can indeed explain how social conflict can be resolved without overt aggression.

## 1. Introduction

In the context of sexual selection, competition among females has been much less well studied than competition among males, but the same is not true in the context of social evolution. Understanding how females compete over reproduction in social groups, and why the outcome of female–female competition varies within and between species, have been major themes of research on inclusive fitness and cooperative breeding for the last three decades [1–5]. The focus on females has arisen in part because of enduring interest in the evolutionary puzzle posed by sterile worker castes in the female societies of Hymenoptera [6,7]; and because of observations of conspicuous forms of female competition in cooperative vertebrates, including mutual egg destruction, infanticide and eviction [8–14]. In social Hymenoptera, for example, reproductive conflict among females arises because workers can often gain from producing male offspring at the expense of the fitness interests of the queen and other workers [15]. Queens and workers respond to this selfish reproductive behaviour by identifying and destroying (or ‘policing’) worker-laid males [16,17]. In social birds and mammals, conflict frequently arises among females over offspring production and may also be resolved through overt destructive forms of competition [8,18,19]. However, in both insects and vertebrate societies, reproductive conflict is sometimes resolved apparently peacefully, prior to the inception of subordinate reproduction, because subordinates abstain completely from any reproductive activity [20–22].

A number of formal theoretical models have been developed to examine how natural selection acts on strategies of reproductive competition in animal societies [1,2,23–25]. These models were originally developed to investigate variation in reproductive skew in ‘cooperative’ societies which feature conspicuous helping behaviour. However, since they examine competition over any shared resource, such as food, territory or mates, the models can be applied with little or no modification to understand competition in non-cooperative social systems, such as those of many primates [26], birds [27], coral-dwelling fish [28–32], insects [33] and arachnids [34]. While early models focused on the causes of inequity in reproductive payoffs, more recently attention has shifted to what the models can tell us about the behavioural mechanisms that animals can use to control each other's behaviour, such as aggression, infanticide and the use of threats [35–38]. For example, a female might produce a larger number of offspring when competing with other females [39]; or she may attempt to kill a rival breeder's offspring [40–44]. She may also wield subtle means of reproductive control, for example, by raising the costs of breeding to other females through harassment [8], or threatening to attack or evict them if they attempt to breed [38,45]. Some of these behavioural mechanisms for resolving conflict (such as aggression) are conspicuous to an observer, whereas others (such as the threat of infanticide or eviction) are more difficult to detect. While our main focus in this paper is on reproductive conflict, understanding how aggression and threats shape social behaviour has broader relevance to the study of sexual selection [46], social hierarchies [38] and population structure [9].

In this paper, we discuss the possible evolutionary causes of these different mechanisms of reproductive control, and review recent theory which helps to understand how female–female competition is manifested in social species. We are particularly concerned to identify conditions and mechanisms which permit reproductive conflict to be settled peacefully, that is, without recourse to destructive or costly acts of harassment, attack, infanticide or forcible eviction. These behaviours can be grouped together as forms of ‘overt aggression’, but may have very different underlying causes. We identify two distinct mechanisms which reduce selection for costly investment in aggression or other acts of violence: (i) destruction competition and (ii) the use of threats. Both these mechanisms are particularly (but not exclusively) relevant to conflict among females, and we focus our empirical attention throughout on female–female conflict. Reproductive conflict models suggest that intrasexual competition among females will have different consequences for selection on morphology and life history compared to males. Our main empirical focus is on social mammals and birds because these are the systems where, for reasons explained below, female reproductive competition seems most likely to be settled without overt aggression.

## 2. Modelling social conflict

Models of reproductive conflict examine conflict over limited resources between members of a social group. The term ‘group’ is here used in a loose sense to refer to systems in which there is local resource competition among offspring [47,48]. We will use the term conflict to refer to situations where the optimal resource allocation for all group members cannot be simultaneously satisfied, so that there is a genetic conflict of interest between individuals with respect to alleles influencing resource acquisition [49]. Almost all models of the evolution of social conflict examine the conflict between two players, or *N* identical players, for reasons of tractability [42]. Here, we focus on two player models, although we consider conflict in larger groups where we expect this to make a qualitative difference to patterns of aggression.

### (a) Conflict resolution

What do we mean by conflict resolution? We define a conflict to be resolved when each competitor behaves in a manner that maximizes its inclusive fitness, given the behaviour of its opponents. That is, we adopt a game theoretical definition (specifically, a Nash equilibrium or, in the case of the sequential model of threats, a subgame perfect equilibrium; [50]) rather than a biological definition of conflict resolution. This differs from Ratnieks *et al.* [51, p. 584], writing about conflicts in insect societies, who define conflict resolution as ‘an outcome that reduces to a low level the proportion of the colony's resources that are wasted in conflict’. Conflicts of interest that result in aggression, fighting, infanticide, eviction and so on are by this latter definition not ‘resolved’, but by our definition these behaviours are part of the resolution mechanism. As our aim is to understand variation in the extent to which potential evolutionary conflict (measured as the disparity in fitness optima between competitors; see below) is manifested as actual overt conflict (aggression), we view both peaceful outcomes (which feature no overt conflict) and non-peaceful outcomes (which do feature overt conflict) as different stable endpoints in the coevolution of conflict strategies. Peaceful conflict resolution (which we try to explain) is therefore a subset of the forms of conflict resolution that can evolve.

### (b) The battleground

**Figure 1.**
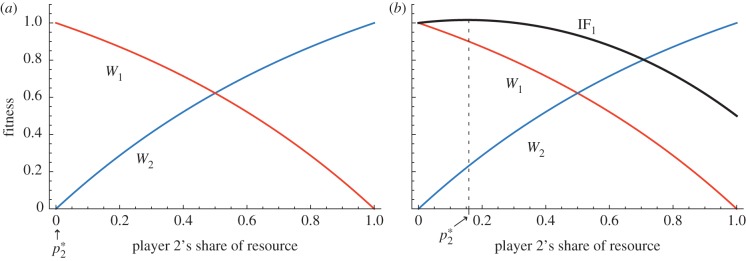
Defining the battleground of evolutionary conflict over limited resources. (*a*) The direct fitness of players 1 and 2 (*W*_1_ and *W*_2_) as a function of player 2's share of a valuable resource, *p*_2_. Player 1's share is 1 − *p*_2_. In the figure, we assume some degree of diminishing returns in the net fitness benefits of increasing resource share. The particular diminishing returns function we use is *W_i_* = *V*[(1−e*^−qp_i_^*)/(1−e*^−q^*)], where the parameter *q* (0 < *q* < *∞*) determines the ‘bowedness’ of the diminishing returns function (*W_i_* approaches linearity as *q* approaches 0; and is highly ‘bowed’, where 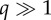). In the case shown *q* = 1. If the two players are non-relatives (as in (*a*)), the optimal division of resources from the perspective of player 1 is 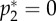, and from the perspective of player 2 is 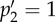. Thus, the zone of conflict or ‘battleground’ in the case of competition between non-relatives is simply all of the available resource. (*b*) The case where the two players are genetic relatives (specifically, in the plot we assume *r* = 0.5). IF_1_ is the inclusive fitness payoff of player 1 (calculated as *W*_1_ + *r W*_2_). The optimum division of the resource for player 1 is the value of *p*_2_ which maximizes IF_1_, i.e. the value which solves the equation ∂IF_1_/∂*p*_2_ = 0. Given our chosen fitness functions, the solution for player 1's optimal allocation is 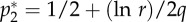; and (since the players are symmetrical) for player 2 the optimal allocation is one minus this expression, i.e. 1/2 − (ln *r*)/2*q*. Thus, the battleground of conflict is 1/2 ± (ln *r*)/2*q*. Note that the value of the resource *V* has no effect on the width of the battleground. The lower and upper bounds of the battleground get closer together as *r* increases and as *q* increases. In other words, increasing relatedness and increasing ‘bowedness’ of the fitness function draw together the fitness optima of competitors, reducing the scope for evolutionary conflict.

To illustrate the potential for evolutionary conflict between social animals, consider two members of a group, player 1 and player 2, who compete for a share of a resource of value *V*. The direct fitness of player *i* is denoted *W_i_* and is an increasing, diminishing returns function of that player's share *p_i_* of the resource (where *p* ranges from 0 to 1). If the two players are unrelated and have no other ‘stake’ or interest in each other's fitness, then the optimal share for each competitor is simply all of the available resource (figure 1*a*). If the two players are genetic relatives, then the optimal share of the resource for each player may be less than 1 (figure 1*b*). In the latter case, the zone of evolutionary conflict (or ‘battleground’) between the two players is reduced. The width of the zone of conflict gives a simple measure of the ‘scope’ or potential for conflict, that is, the degree to which the interests of the two players diverge. In addition to genetic relatedness, factors such as reciprocity, strong intergroup competition and other forms of mutual ‘interdependency’ [52], can draw together the fitness optima of the competitors and so reduce the scope for evolutionary conflict.

However, the width of the battleground does not necessarily predict the intensity or frequency of overt acts of aggression. What matters is the inclusive fitness returns of effort invested in aggression, which in turn depends on how aggression is assumed to ‘work’. Do animals use aggression directly to extract or appropriate resources from rivals? Does aggression function as a signal to deter rivals from challenging for control of a resource? Is aggression observed only when more subtle forms of social control have failed? To make testable predictions about patterns of social aggression, it is necessary to make an explicit assumption about its precise function, and how players respond to each other's aggressive acts.

### (c) Conflict strategies: sealed bid or behavioural negotiation?

A fundamental distinction between different conflict resolution modelling approaches is whether they examine the coevolution of ‘fixed’ genetic strategies of conflict, or whether they allow for a degree of real-time behavioural responsiveness or ‘negotiation’ [53–55]. The former types of model are called simultaneous or ‘sealed bid’ models because they assume that the effort invested in conflict is not sensitive to the precise behaviour of a social partner. Individuals commit to a level of aggression (or conflict effort) and stick to it. Models of negotiation, by contrast, assume that players can observe and respond to each other on a behavioural time scale. In these models, one or both players may adjust their level of conflict effort after observing the effort of their partner.

At their heart, these two types of modelling approaches examine two different ways that natural selection can act on conflict behaviour, but they are not necessarily alternatives. The level of aggression of a particular individual animal may be due in part to selection acting on a baseline ‘fixed’ genetic component, which determines average level of aggression irrespective of the particular social partner with which she is paired; and selection for behavioural responsiveness, which determines how that baseline level is adjusted according to the particular reactions or phenotypic features of that partner. Sealed bid models may therefore be particularly useful to understand the evolution of aggressive or non-aggressive ‘personalities’ and developmental commitment to weaponry, badges of status or other morphological traits that are important in conflict, such as body size. On top of this (and perhaps linked to sealed bid commitments in a way that has yet to be investigated), negotiation models provide a way to understand social ‘sensitivity’, and the varied forms of dominance interactions, rituals and signals that are widely observed in animal societies, but remain quite poorly understood.

We first describe a sealed bid approach to our two player model of social competition, and then outline a sequential (or ‘threat’) model which incorporates behavioural responsiveness into the framework in a simple way. For both the sealed bid and sequential cases, we focus on the conditions where conflict can be resolved with little or no sign of overt conflict. The models make testable predictions about the genetic and ecological conditions that can lead to peaceful conflict resolution. In the discussion, we compare these predictions with empirical data and suggest ways in which the main shortfalls in current knowledge can be addressed.

## 3. Sealed bid models of reproductive conflict

In this model, we assume that player 1 can invest effort *x* in costly competitive acts to shift the resolution of conflict towards its own optimum. Player 2 can invest effort *y* to do likewise. In evolutionary (and economic [56–58]) models of conflict, the function that specifies how effort invested in conflict translates into relative success in competition is called the contest success function and will be denoted *F*. The relative success of player 1 is *F*_1_ and that of player 2 is *F*_2_ (where *F*_1_ + *F*_2_ = 1).

As we are interested in social competition, we assume that effort invested in conflict decreases the value of a shared resource, or the productivity of a group. Thus, the value of the resource *V* is a declining function of effort levels *x* and *y.* In the simplest case, we assume that there are no other personal fitness costs of conflict; including these costs does not in any case qualitatively affect the results on which we focus [42]. With these assumptions, player 1's direct fitness is3.1
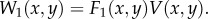


How is effort invested in conflict likely to convert into success? In other words, what form should we assume for the contest success function *F*? Almost all models of evolutionary conflict assume that this function takes the form of a ratio, with a focal individual's effort as the numerator and the mean or the summed efforts of all competitors as the denominator [42]. These conflict models are called ‘ratio form’ models [56,59]. For example, two widely known ratio form models of social conflict are Reeve *et al*.'s [41] tug-of-war model and Frank's [40] model of policing. In Reeve *et al*.'s model, player 1's relative success is given by the ratio *x*/(*x* + *by*), where *b* (<1) allows for an asymmetry between players 1 and 2 in ‘strength’ or the efficiency with which effort invested in conflict converts into reproductive success. In Frank's [40] *N*-player model, the relative success of player *i* is 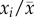, i.e. the success of player *i* depends on their effort relative to the mean effort of all players.

One of the most important features of ratio form models is that no evolutionarily stable (ES) equilibrium exists where both players invest zero effort. In other words, ‘peaceful’ outcomes of evolutionary conflict are not predicted in this type of model. To see why, imagine a two-player ratio form model in which both players invest zero effort. This cannot be an ES equilibrium because any mutant player who invested an infinitesimally small level of effort would win all the resource. The marginal gains of investment at *x* = 0, *y* = 0 are infinite, and so a situation of mutual peace is unstable. A situation of one-sided peace, where one player invests zero and the other invests positive effort, is also not a stable equilibrium outcome in ratio form models [42]. The conclusion from ratio form models is that players get zero payoff if they do not invest in conflict, and so any disparity in the optima of competitors is always manifested as actual conflict, for example, via overt aggression.

### (a) How is peaceful resolution possible with sealed bids? Production versus destruction competition

Some types of biological competition, however, do not fit the assumption of ratio form models. Cant [42] argued that there are two distinct forms of competition that can occur among social partners: ‘production’ and ‘destruction’ competition. (Note in Cant's original paper, the latter form of competition was called ‘suppression’ competition, but since suppression has been used widely to describe any downregulation of female fertility, socially imposed or not [60–62], we will use the more specific term destruction competition from now on.) Production competition involves maximizing the proportion of competitive efforts or units produced (e.g. offspring); destruction competition involves eliminating or destroying the competitive units produced by others. To illustrate the distinction, consider an example of two female birds laying eggs in a shared nest [42]. If competition takes the form of a scramble among chicks for food, then a female's fitness will be determined by her proportional representation in the communal clutch. This is an example of production competition, and a ratio form model would capture the payoffs of producing extra offspring to maximize success in conflict. If on the other hand competition takes the form of egg destruction or infanticide after the clutch is produced, a female that invests nothing in conflict need not obtain zero payoff, particularly if it is difficult for an infanticidal female to accurately discriminate the maternity of offspring. In this case, the two females are engaged in destruction competition. In destruction competition, non-investors in conflict can still gain a positive fitness payoff, so it may pay to accept the status quo division of resources rather than investing in destructive behaviour. Note that while destruction competition can be applied intuitively to reproductive conflict among females, it is less clear how well it applies to intrasexual conflict among males. Males typically compete for a share of paternity of offspring rather than via the production or destruction of competitive ‘units’ *per se*. However, destruction competition may apply well to strategies of intersexual conflict such as mate guarding, harassment or the production of seminal toxins [42,63,64].

How can we model destruction competition? Conveniently, we can capture the main characteristics of destruction competition using a simple alternative form for the contest success function, in which relative success depends on the difference between the players’ conflict efforts, rather than the ratio of their efforts. In a difference form model, zero investment in conflict does not necessarily bring zero payoff, the distinctive feature of destruction competition that we want our model to possess. In fact, the economist Skaperdas [58] has shown that the ratio and difference forms are the only two functional forms which possess all of the appropriate properties that we want in a contest success function (for example, that *F_i_* is an increasing function of the effort of player i; and that *F*_1_ + *F*_2_ = 1). Thus, in mathematical as well as biological terms, contest success functions divide naturally into these two forms.

Cant [42] analysed a model based on fitness expression (3.1) but using the following difference form contest success function, adapted from Hirshleifer [56]:3.2
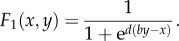


**Figure 2.**
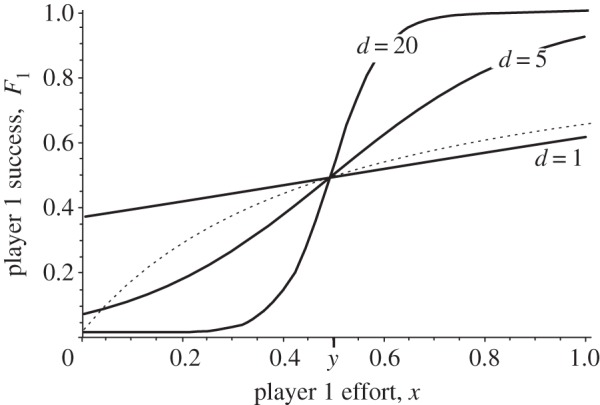
Contest success in a difference form model of evolutionary conflict. We plot the relative success of player 1 as a function of her effort invested in conflict *x* (using the difference form contest success function (3.2) in the text), for three values of the ‘decisiveness’ parameter *d*. Player 2 is assumed to invest *y* = 0.5. The effect of the decisiveness parameter is to change the marginal benefits of superior effort invested in conflict. Where decisiveness is high, a small advantage in conflict effort converts to a large advantage in relative success. For comparison, the dotted line shows the ratio form contest success function (*x*/(*x* + *by*)) used in Reeve *et al*.'s [41] tug-of-war model. In difference form models, unlike ratio form models, player 1 can still obtain some success even if she invests nothing in the conflict. Other parameter: *b* = 1.

It is easy to see from the denominator that what matters here is the difference in the players' efforts. The parameter *d* ranges from zero to infinity and is called ‘decisiveness’; it scales the marginal gains of superior effort invested in conflict. The shape of this function for three values of the decisiveness parameter *d* is shown in figure 2. In a difference form model, a player who invests nothing in conflict can still gain some reward.

**Figure 3.**
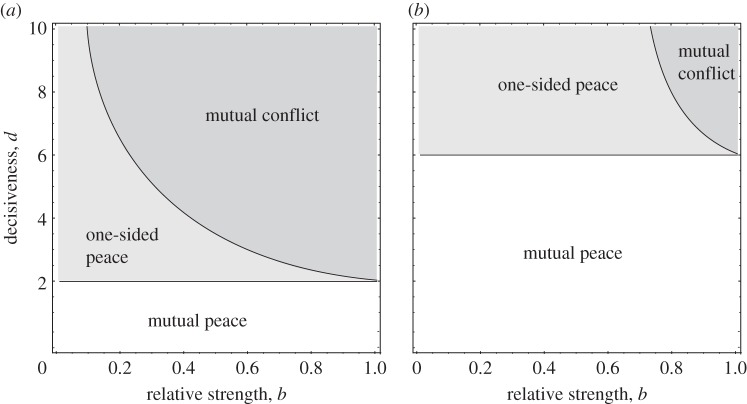
Peaceful and non-peaceful outcomes in a model of ‘destruction’ competition (adapted from [42]). The figures show the three types of ES outcome that can arise when we vary the two parameters of the contest success function (3.2), decisiveness *d* and relative strength or efficiency *b*. In the ‘mutual peace’ zone, the ES outcome is for both players to invest zero conflict effort. In the ‘one-sided peace’ zone, only the stronger player invests positive effort in conflict. In the ‘mutual conflict’ zone, the ES outcome features positive conflict effort by both players. (*a*) The case where players are non-relatives. In this case, the ES outcome is mutual peace below a threshold level of decisiveness equal to 2. (*b*) The case where the players are related by coefficient 0.5. In this case, mutual peace is the ES outcome below *d* = 2(1+*r*)/(1−*r*), i.e. *d* = 6. The ES outcomes are mutual peace in these zones because for these values of decisiveness and relative strength, investment in conflict effort is just not profitable.

Figure 3 shows the results of the difference form model analysed in [42]. The model predicts that high relatedness, asymmetry in strength and low decisiveness all promote peaceful resolution of conflict. Relatedness has a dual effect: it draws together the fitness optima of competitors and also reduces the profitability of effort invested in conflict. Asymmetry in strength promotes one-sided peace in which only the stronger individual needs to invest in conflict to keep the weaker individual peaceful and submissive. Finally, below a threshold level of decisiveness, *d*_crit_, the outcome is zero conflict effort, despite much potential for conflict. For the model shown in figure 3, this critical level of decisiveness is given by a simple expression
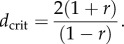

Thus, the model predicts that high relatedness and low decisiveness allow evolutionary conflict to be resolved without any costly or wasteful acts of conflict, for the simple reason that in these circumstances conflict effort does not pay. In biological terms, we can interpret decisiveness in a number of ways. Decisiveness is high in contests of endurance (e.g. a war of attrition; [65]), since an individual needs only to invest slightly greater effort than her opponent to claim all of the resource. At the other end of the scale, decisiveness is low where the outcome of fights has a large stochastic component, or where a non-investor in conflict can inherit the resource in the future (as in social queues; [66]). We can also expect decisiveness to be low where there is a high degree of uncertainty about relative strength or how effort invested in conflict converts to success. The take home message is that where competition involves destruction competition, a number of biologically plausible factors can render investment in aggression unprofitable, so that the stable outcome is peace even among social partners with very different interests.

## 4. Sequential (or ‘threat’) models of reproductive conflict

The sealed bid approach described above assumes that, at the ES equilibrium, the population consists of pairs of identical player 1 ‘types’ and player 2 ‘types’ who invest ES efforts x* and y*, respectively. There is no other individual variation in underlying quality or strength, so there is no need for players to observe and be responsive to changes in each other's behaviour [53,67,68]. The situation is different if individuals vary, and we allow players to observe and respond to their opponent's behaviour. In this case, the sealed bid solution may no longer be ES [53]. Natural selection can favour individuals that negotiate before or during a competitive interaction, and favour ES levels of ‘responsiveness’ to the effort of an opponent or social partner, rather than ES fixed efforts [53].

The simplest way to incorporate responsiveness of this type is to move away from the assumption of simultaneous sealed bids, and instead assume that the interaction consists of a sequence of two steps: player 2 claims a share *p* of the resource, which is observed by player 1 before she decides on her response. This sequential structure opens the door to the use of threats to influence behaviour. Thus, player 1 may threaten to inflict a cost on player 2, if player 2 claims more than a threshold share of the resource *p*_crit_. For simplicity, we will assume in this model that only player 1 has the power to exercise a threat, so in this respect she can be thought of as socially dominant to player 2. We further assume, for simplicity, that player 2's claim on the resource is cost free, unlike the sealed bid model above in which the share (or probability of winning, in the case of an indivisible resource) of player 2 depends on how much she and her opponent invest in costly conflict. The assumption of cost-free claims is not critical to our arguments: Cant & Johnstone [45] show how to build a ‘synthetic’ model which incorporates sealed bids and threats in the same framework. In their model, shares of the resource are determined by a first step in which players invest sealed bid conflict efforts *x* and *y*, but both players can respond by exercising a threat to break up the group if their share of the resource drops below a threshold level.

**Figure 4.**
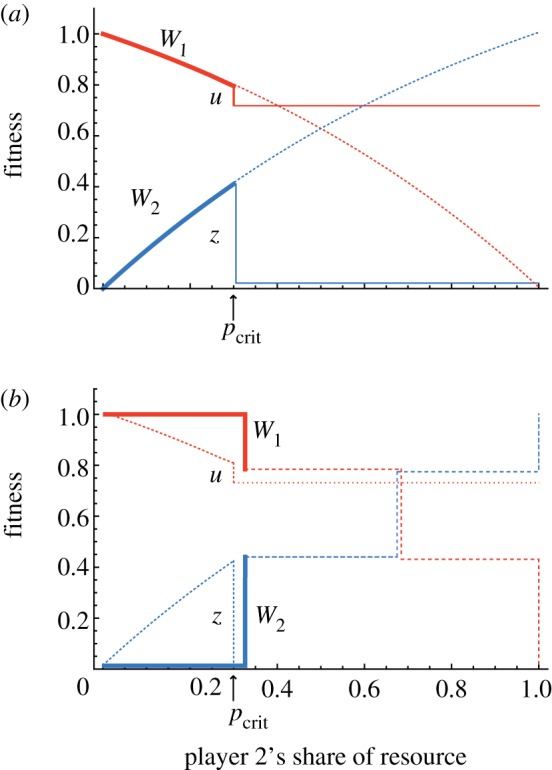
Fitness payoffs in a sequential or threat model of resource competition. In this model, player 2 makes a first move by claiming share *p*_2_ of the resource. Player 1 observes player 2's behaviour and can respond by inflicting a punishment (for example, killing her offspring) if the claim exceeds a threshold level *p*_crit_. The punishment costs player 1 *u* fitness units to carry out, and for simplicity is assumed to reduce player 2's fitness to zero when triggered. Bold lines represent stable outcomes where the threat of punishment is not exercised; thin lines represent the fitness consequences for both players when the threat is triggered. Dotted lines and dashed lines represent underlying fitness functions (dotted lines: the case where the resource is continuously divisible; dashed lines: the case where the resource is discretely divisible). If player 2 knows (or believes) with certainty the location of *p*_crit_, her best strategy is to claim up to this threshold and no more. The threat of punishment by player 1 in the second step forces player 2 to exercise restraint in the first step. (*a*) The case where the resource is infinitely divisible and hence the fitness functions are smooth curves. (*b*) The case where player 2's claims come in discrete chunks or packages (such as individual offspring). Specifically, in (*b*) player 2 can only make claims of one-third of the resource at a time, resulting in a stepped fitness function for both players. In this situation, player 2's minimum claim is one-third, which exceeds the threat threshold *p*_crit_, so player 1's threat is sufficient to enforce a monopoly on the resource. In general, the more ‘bulky’ the discrete packages of resource, the more player 2 should err on the side of caution, and the further below *p*_crit_ we can expect the resolved division of resource to lie.

Player 1's threat can deter player 2 from claiming more than *p*_crit_ if player 2 ‘believes’ that player 1 will in fact carry through on this threat, i.e. if the threat is credible (figure 4*a*). Credibility is a key issue here because exercising a threat (i.e. actually punishing player 2) may involve some cost *u* to player 1, so the question arises as to why player 1 would act in a way that involves an immediate cost to her fitness. There are a number of biological mechanisms that can render a threat credible, despite a cost *u*. First, even in a one-shot interaction, player 1 could be selected to carry out the costly punishment if player 2's claim on the resources is so far over *p*_crit_ that the costs of allowing the claim are greater than *u*. Second, in repeated interactions the cost *u* may be repaid later because carrying out the threat trains player 2 to exercise more restraint in subsequent rounds of the interaction. Third, if the threat is an ‘exit threat’, such as threatening to leave or evict the other player, the threat can be credible if it allows player 1 to avoid further exploitation and assort with a more cooperative partner in future (i.e. one that claims less than *p*_crit_) [69]. Note that in each case the cost *u* is repaid, so all of the threatened actions are forms of ‘self-serving’ punishment [69]. Raihani *et al.* [70] prefer to restrict the term punishment to cases where the victim pays back the cost *u* (i.e. mechanism 2 above); and to refer to punitive acts as ‘sanctions’ when the cost *u* is repaid in other ways (e.g. mechanisms 1 and 3 above).

When behaviour is shaped by threats, the degree to which competition is manifested as overt aggression or acts of conflict depends on the effectiveness of the threat at inducing pre-emptive restraint or cooperation in a social partner. Where a threat is effective it rarely needs to be carried out, so effective threats can resolve conflict with little or no overt sign of aggression. Two examples help to illustrate this point. First, some coral-dwelling fish form regular size hierarchies with a more or less constant size difference between ranks [28,30–32]. Experiments have shown that the threat of eviction causes a subordinate individual to stop growing before it gets too close in size to its immediate dominant ahead of it in the hierarchy. The threat is very effective indeed because smaller, subordinate fish do not overshoot the threshold to trigger eviction, so actual evictions are extremely rare in nature. Contrast this situation with cooperatively breeding banded mongooses, where multiple dominant and subordinate females give birth in each breeding attempt [9]. Dominant females respond to reproductive competition from subordinate females by aggressively evicting groups of subordinates, with pregnant subordinates being specifically targeted for eviction. However, the threat of eviction is not effective at inducing pre-emptive reproductive restraint in subordinate females, so aggressive, conspicuous evictions are commonly observed in this system.

### (a) Threats and information

Where there is complete information on both sides, player 2 will claim up to *p*_crit_ and no more, and the threat will never be exercised. This provides one explanation for how conflict can be resolved without any observable aggression. A more interesting (and more realistic) situation arises if the players have incomplete information about key aspects of the competition. Two types of uncertainty can be expected to affect the degree to which conflict is manifested as aggression: (i) uncertainty about the location of threat thresholds and (ii) uncertainty about the target of punishment. The latter form of uncertainty is particularly important for threats of policing or infanticide, and for threats directed against multiple recipients.

Considering the first type of uncertainty, player 1's threat can only be effective if player 2 knows where the threat threshold lies, i.e. the value of *p*_crit_. In a repeated interaction, this information could be gained from trial-and-error learning, that is, player 2 could make a given claim *p′*, and, depending on whether the threat is triggered, raise or lower this claim in an attempt to approach the threshold *p*_crit_. This kind of trial-and-error learning is more likely to occur where the impact to player 2 of triggering player 1's threat is small (i.e. the quantity *z* in figure 4*a* is small). For example, in insect societies, the impact to a reproductive worker of losing an egg is very small, so threats of egg destruction are unlikely to deter workers from breeding. Indeed, in some social wasps and bees, a proportion of workers produce eggs even when these are almost certain to be destroyed [17]. By contrast, in mammals, the cost of gestation and lactation mean that losing an offspring to infanticide has a much larger impact on lifetime fitness, so triggering a threat is a costly method to gain information about the location of a threat threshold. If a subordinate female mammal or bird decides to make any claim on the resource, it would be best to start small and build up from there, in the same way that a growing subordinate clownfish [32] or goby [28,29] approaches the size of its immediate dominant from below.

An alternative means to reach a behavioural settlement without triggering the threat is for both parties to communicate, to establish the location of the threat threshold. However, since player 1 has an incentive to exaggerate her willingness to exercise a threat, signals by player 1 conveying information about the location of the threshold may need to be costly to ensure their honesty [54]. For example, costly but relatively harmless dominance displays that are widespread in animal societies could plausibly function to advertise the location of thresholds of more violent acts, such as attack and infanticide [38,45]. Recently, Szamado [71] (building on a model by Enquist [72]) showed that the type of threat signals that are most likely to evolve are those for which the main cost of the signal is an increased risk of provoking an escalated fight. This mechanism for ensuring honesty of threat signals was first recognized by the political economist Schelling [73], who gave it the catchy title ‘the threat that leaves something to chance’. These models of threat signalling may explain why dominance displays often involve mounting, charging or lunging at a subordinate, rather than traditional ‘handicaps’. It is an intriguing and counterintuitive idea that aggressive and provocative acts among animals may actually serve to keep the peace and maintain the stability of social groups. It also illustrates why defining conflict resolution in terms of the absence of costly aggression (e.g. [51]) might inadvertently distract attention from some of the most interesting and poorly understood processes by which animals settle their differences.

The second important form of uncertainty arises when there is more than one potential target for punishment when *p*_crit_ has been exceeded. For example, suppose that the punishment involves killing player 2's offspring rather than attacking player 2 herself. Cant *et al*. [74] analyse a sequential infanticide game in which there are two players who produce one offspring each. If player 2 breeds, player 1 can choose to kill one offspring. However, her discrimination might not be perfect: with probability *s* player 1 kills the offspring of player 2, with probability (1 – *s*) she kills her own offspring. If both females breed and raise two offspring, each player obtains a fitness payoff of 1 − *k* (where *k* < 1) to reflect competition among the offspring. Cant *et al*. [74] show that in this model, infanticide occurs when discrimination lies in the intermediate range 1 − *k* + *c_i_* < *s* < 1 − *c_b_*, where *c*_i_ is the cost to player 1 of carrying out infanticide, and *c_b_* is the cost to player 2 of producing an offspring. If *s* lies below the lower bound, the threat of infanticide is not credible: player 2 will certainly breed and player 1 will refrain from infanticide because her ability to distinguish the maternity of offspring is too poor. If *s* lies above the upper bound, the threat of infanticide is sufficient to deter player 2 from reproducing in the first place, so again no infanticide will occur. Note that as offspring become more costly (i.e. as *c_b_* increases), as the cost of competition diminishes (i.e. as *k* decreases) and as the cost of infanticide grows (i.e. as *c_i_* increases), the zone of infanticide shrinks. The model predicts that variation within and across species in the costs of cobreeding (*k*) and the costs of producing offspring (*c_b_*) determine the extent to which threats can be effective at suppressing reproduction among subordinates and the ease with which subordinates can escape from suppression (for example, by scrambling cues to maternity [14,75]). Because the cost of producing offspring is generally much higher in vertebrates compared with insects, there is greater potential for policing by threats rather than acts of infanticide in the former than the latter [74].

The ability to target threats accurately plays a similar role in determining the effectiveness of threats in groups greater than size 2. In a pairwise interaction (such as that illustrated in figure 4), a female who claims too large a share of the resource is certain to suffer the impact when the threat is triggered. In larger groups, however, it may be difficult for a threatening individual to determine which individual to punish. If a dominant individual does not dish out punishments accurately, the effectiveness of the threat of punishments breaks down because transgressors may escape punishment, and non-transgressors may end up being punished by mistake. For this reason, in larger groups, threats are most effective where there is a clear hierarchy or chain of command, so that the group is broken into a series of dyads [38]. When each individual monitors and punishes one other in the group, threats of punishment can stabilize cooperation without needing to be exercised. Again, fish size hierarchies provide the best biological example: threats are targeted at an individual one rank lower in the hierarchy, and a growing subordinate is sure to suffer the consequences of its own actions. It is a long-standing hypothesis in the ethological literature that dominance hierarchies stabilize groups, because subordinates can use information from signals and previous encounters from those above them to avoid repeatedly entering into unprofitable fights with higher ranked individuals [76,77]. Our focus on threats versus acts provides a new insight into why dominance hierarchies help to reduce the frequency of costly overt conflict in multi-member groups: they increase the targeting accuracy of threats.

## 5. Discussion

In this paper, we have outlined two distinct theoretical mechanisms that might explain why evolutionary conflict need not necessarily be manifested as overt aggression. The models differ in their assumptions about whether individuals adjust their behaviour according to the behaviour of their competitor, i.e. their degree of social sensitivity or responsiveness. One of the points we wish to emphasize, however, is that while there is a dichotomy in the game theory literature between these two modelling approaches (where sealed bid and sequential models are referred to as ‘strategic form’ and ‘extensive form’ games, respectively [50]), in biological reality no such dichotomy need exist. Aggression is a catch-all term for what is a complex and enormously variable behaviour, which may serve a number of different functions, perhaps simultaneously. For example, a dominant female may attack a rival female to signal that she is still strong, but this attack might also weaken the victim and induce stress which suppresses her ovarian function. We also usually do not know (without performing an experimental manipulation) how responsive individuals are to changes in each other's aggression levels. Faced with this rather daunting wall of ignorance, a useful way forward is to build simple explicit models which we can hope capture the main biological features of social competition, and use them to make testable predictions about conflict investment and the conditions favouring peace. Inevitably, specific tests of the predictions and assumptions of different models take time to carry out, so in the meantime the best we can do is to see whether the models provide insights into systems for which there are good existing data.

Of the two modelling approaches described above, which model or combination of models applies best to a given biological system depends on the degree to which it pays to be socially sensitive. At one end of a continuum, we can imagine situations where there is no selection for behavioural responsiveness because the consequences of claiming a resource, or producing offspring, are highly predictable. For example, in colonies of naked mole rats, subordinate females never reproduce in the presence of the dominant female (or ‘queen’). Let us hypothesize that this is because a subordinate that reproduces is guaranteed to suffer a net fitness cost from breeding, owing to the dominant's capacity to kill her young. In these circumstances, negotiation is not required for the subordinate to make an adaptive decision to exercise reproductive restraint, and so there would be no selection for fine-scale behavioural tuning of reproductive behaviour in response to the credibility of the threat of infanticide. As subordinate females may simply be hard-wired to inhibit ovarian function in the presence of the queen, we could in principle use a sealed bid model to try to understand why the outcome of reproductive conflict is a complete lack of subordinate reproduction and the consequent monopolization of resource by the dominant. Our destruction competition model suggests that this is an example of one-sided peace, as only the dominant invests any effort into maintaining her monopoly [78], and that the main drivers of this pattern are the high asymmetry in strength and high level of genetic relatedness between females. At the other extreme, low reproductive skew and mutual peace among female lions may be explained by the possession of potentially lethal weaponry (a non-negotiable feature of the conflict). Fighting between lions is extremely risky (in terms of injury) for both sides, so it may be better to refrain from any aggressive acts aimed at securing a greater share of the reproductive output of the group. In the destruction competition model, this corresponds to a situation in which decisiveness is low, so mutual peace can be an ES outcome even among unrelated females with much potential for conflict.

### (a) The role of threats in the evolution of peaceful reproductive restraint

As we demonstrate above, where dominants are sufficiently capable of disrupting or punishing subordinate reproduction, selection may favour the evolution of pre-emptive reproductive restraint among subordinates given the threat of such action [38,44,45,79–84]. As subordinate restraint may then obviate the need for dominants to act upon their threats, this provides a potentially general mechanism for the peaceful resolution of reproductive conflict [38,44,45,79–84]. Such peaceful conflict resolution via threats has important implications, not only for understanding the incidence of overt aggression in societies. From a practical perspective, where subordinates exercise complete reproductive restraint this can markedly hamper attempts to identify the ultimate causes of their quiescence, as any threats that effectively enforce restraint may rarely need to be actioned [21,38,44,80,84–86]. From an evolutionary perspective too, where reproductive conflict is resolved without overt escalated contests this may markedly relax intrasexual selection for the exaggeration of traits that yield success in such contests ([46]; see also below). Threats may ultimately prove to play a pervasive role in reproductive conflict resolution [38,44,80,82,87], but while reproductive abstention does appear to be widespread [21,80,85,87–94], there remains little compelling evidence that it actually is enforced by threats [21,38,44,80,84,85]. This is doubtless due in part to the logistical and ethical challenges entailed in experimentally inducing reproductive transgressions so as to elicit the threatened actions that may usually prevent them ([38,80]; see [28,29] for the induction of growth restraint transgressions in fish). It is clear, however, from widespread evidence of overt destructive competition among females [5,9,18,60,80,81,95–100] that the threat of action alone is not always sufficient to yield reproductive restraint. Attempts to understand the incidence of peaceful conflict resolution demand therefore that we consider the causes of variation in the effectiveness with which threats yield restraint.

### (b) Impact, accuracy and perception

In order for dominants to induce reproductive restraint using threats, they must first be able to detect subordinate reproduction or the fertility that underpins it [80]. In scenarios where subordinate reproduction is actually impossible to detect, dominants might conceivably still impose reproductive restraint if they were capable of detecting fertility *per se* (prior to reproduction) and disrupting or punishing it instead [80]. Where subordinate reproduction or fertility are detectable, at least three factors will determine the effectiveness with which threats of action by dominants induce reproductive restraint [38]: (i) the impact on the fitness of the transgressor of being subjected to the threatened action, (ii) the accuracy with which the action can be directed at the transgressor and (iii) the perception of the threat by both parties (the level of information available regarding the consequences of the threat and when it will be triggered).

The impact of a threatened action may be insufficiently high to induce restraint either because of inherent limitations of the action itself (e.g. temporary evictions in meerkat and banded mongoose societies do not always disrupt subordinate pregnancies [9,96]) or because dominants suffer costs when executing the action that constrain their ability to do so. For example, dominant female meerkats appear to suffer constraints on their ability to evict their subordinates when in poor condition themselves and when there are simply too many of them [96]. Indeed, evidence from banded mongoose societies supports the view that such evictions are costly to enforce [101]. The impact of actions that terminate entire reproductive attempts may also be lower in species that breed in frequent low-cost attempts (e.g. egg laying in insects) rather than infrequent high-cost ones (e.g. pregnancies in mammals).

Dominants may also suffer constraints on the accuracy with which they can target either reproductive transgressors or their young, potentially due in part to selection for counterstrategies among subordinates [14,102]. As the targeting accuracy of any threatened action declines, two key effects are likely to relax selection for restraint [9,38,80,86,103,104]. First, transgressors are more likely to escape punishment. Second, targeting errors are more likely to erode the incentive to exercise restraint, as individuals that do so may be punished regardless [103,104]. For example, the lack of complete restraint among subordinate female meerkats could be attributable in part to non-pregnant subordinates frequently being evicted alongside pregnant ones ([8], see also [9]). Such indiscriminate aggression may still pay off for dominants given the extreme costs that can arise from failures of suppression (pregnant subordinates are infanticidal [96]). Any factors that facilitate the identification of transgressors and their young, such as small group sizes, dyadic interactions in hierarchies or inescapable chemical signatures of reproduction, may thereby facilitate the evolution of peaceful restraint via threats [38,86]. Indeed, actions with low impact but high accuracy (e.g. the weak punishment of known transgressors) may be just as effective at yielding restraint as those with high impact and low accuracy (e.g. the strong punishment of transgressors subject to identification errors).

Finally, the information available to both the dominant and subordinate about the scale of the threat and, most importantly, the threshold level of reproduction that will trigger it (that is, the value of *p*_crit_ above) may markedly impact whether threats induce restraint [38]. Whenever *p*_crit_ = 0 (i.e. the dominant takes action whenever a subordinate breeds) this may greatly facilitate the evolution of restraint, as simple fixed-response rules could conceivably evolve whereby subordinates invariably exercise restraint without a need for behavioural negotiation (e.g. [105–109]). As reproductive output in reality is packaged into discrete units, a similar outcome might be expected whenever *p*_crit_ is smaller than the minimal unit of reproductive output that a subordinate could produce (figure 4*b*). Given the high incidence of cooperative societies with complete reproductive skew, scenarios where *p*_crit_ ≈ 0 and threats are effective (so simple rules for restraint have evolved) might frequently account for the peace that characterizes such societies [21,80,82,84,87,90].

By contrast, when *p*_crit_ > 0 the situation becomes markedly more complicated mechanistically, as breeding without triggering punishment would now demand the ability to both establish *p*_crit_ and tailor reproductive output accordingly. Where reproduction is achieved via frequent small investments, as is often the case for egg laying in social insects, for example, this might conceivably be achieved as envisaged in the negotiation model above, with workers incrementally adding eggs to the queen's brood until threatening signals are received (indicating that *p*_crit_ is being approached and that egg laying should cease). Similarly, the incremental nature of growth may have facilitated the evolution of growth restraint in response to threats in fish size hierarchies [28,29]. However, a comparable process is more difficult to envisage in reproductive conflict in mammals. As offspring cannot simply be added incrementally to a communal litter, this may preclude subordinates from detecting *p*_crit_ before they have already exceeded it. Even where *p*_crit_ is sufficiently large that one subordinate could breed, where multiple subordinates are present selection may favour a lack of restraint by many [9], particularly if a lag between the inception of reproduction and the production of young (as is the case with pregnancy) yields uncertainty regarding the ultimate output of others. Finally, further complication will arise when *p*_crit_ varies over time. For example, dominant female meerkats evict subordinates and kill their young when pregnant themselves (*p*_crit_ = 0), but tolerate subordinate reproduction when not pregnant (*p*_crit_ > 0) [8,85,96,110]. Selection may thereby favour subordinate females who conceive when the dominant is not pregnant [85], but they must do so lacking information on whether the dominant will have become pregnant (*p*_crit_ = 0) or not (*p*_crit_ > 0) by the time the subordinate gives birth. Such inescapable uncertainty regarding the future value of *p*_crit_ (complicated further by subordinates also becoming infanticidal when pregnant [96]) doubtless underpins the frequency with which subordinate reproduction culminates in evictions and infanticide [8,80,85,96,110]. Indeed, in species where subordinate reproduction is sometimes tolerated (*p*_crit_ > 0) but subordinates cannot incrementally add offspring to the dominant's brood or guarantee breeding asynchronously, the informational challenges entailed in determining *p*_crit_ (or in some cases predicting it) may frequently preclude the peaceful resolution of conflict via threats.

### (c) Integrating threats with the non-punitive factors that may favour exercising restraint

While theoretical models of reproductive sharing have typically considered it a product solely of conflict between dominants and subordinates [2,4,88], empirical research has long highlighted the importance of processes other than rank-related interactions in generating reproductive skew, which could limit the relevance of theory to real world societies. Here, we stress that in order to fully understand the circumstances under which threats precipitate the peaceful resolution of reproductive conflict via restraint we must take an integrated view of the suite of factors that may act in concert to favour restraint. Broadly speaking, the point at which it is adaptive to exercise restraint may be impacted by any factors that reduce a subordinate's expected payoff from breeding, not solely the threat of action by dominants. Such factors have been reviewed elsewhere [80], but, in addition to threats of action, will include two major classes of what we might usefully term ‘non-punitive’ mechanisms. First, several factors may reduce a subordinate's expected payoff from breeding regardless of the presence of the dominant, such as a lack of access to unrelated mates or simply being young, reproductively inexperienced or in poor body condition [60,80,85,92,94,111–113]. Second, a subordinate's expected net payoff from breeding may also be markedly reduced when the dominant is also breeding, regardless of any threat of interference [80,114], for example, due to competition between her own young and those of the dominant [114–119] or the need to kill some of the dominant's young to favour her own [12,19,96].

Appreciating that any threat of action by the dominant will act in concert with such non-punitive mechanisms to devalue a subordinate's expected payoff from breeding, is central to understanding when threats will be sufficiently effective to yield reproductive restraint. This is because when such non-punitive mechanisms strongly devalue a subordinate's expected payoff from breeding, even weak threats of action may be sufficiently effective to induce restraint. Specifically, the threshold levels of threat impact, accuracy and perception (see above) that are required to induce reproductive restraint will be markedly reduced. Placing this in the context of restraint models (e.g. [79] and see above), such reductions in a subordinate's expected payoff from breeding (due to non-punitive mechanisms) might conceivably facilitate the emergence of restraint by (i) reducing the subordinate's own optimal reproductive output (in the absence of any threat of action by the dominant), thereby narrowing the zone of conflict and/or (ii) leaving the subordinate's own optimal reproductive output (and hence the width of the zone of conflict) unchanged, but leaving the subordinate less resistant to reductions in its reproductive output because it values each unit less. In many cases, non-punitive mechanisms may act so strongly as to render it unprofitable for subordinates to breed regardless of the actions of the dominant (as can be the case with inbreeding avoidance, for example [61,89,94]), in which case they will have effectively eliminated the zone of conflict as the subordinate's own optimal output would now be zero, obviating the need for threats.

Recognizing that the extent to which threats yield peaceful restraint will depend upon (i) their impact, accuracy and perception and (ii) the extent to which reproductive payoffs are also devalued by non-punitive mechanisms, may allow one to predict differences between taxa or classes of individual in their propensity to resolve conflict peacefully. For example, we echo Young *et al*. [46] in suggesting that various fundamental aspects of the reproductive biology of females may leave females differentially pre-disposed to exercising reproductive restraint. First, as reproduction by females may often be easier to detect than that by males (while matings may easily go unnoticed, female reproduction may also entail conspicuous oestrus, pregnancy, nest creation and the production of additional eggs and young), reproduction by females may often be more readily disrupted or punished. Second, females may also be better able than males to discriminate their own young from those of competitors and to direct infanticide accordingly [18,86,96], rendering the accuracy of this high-impact action higher among females than males. Finally, subordinate females might also be expected to exercise complete reproductive restraint in response to weaker threats of action from their dominants than males would, because the costs of attempting reproduction in the first place are typically higher for females than males [80,81,120,121]. If such factors do indeed pre-dispose females to exercising restraint from challenging their dominant's monopoly, this might explain why females in cooperative vertebrate societies often show both higher reproductive skew and longer dominance tenures than males, and thereby higher variance in reproductive success [46,121–123]. Indeed, that much of the elevated variance in reproductive success among females could be attributable to peaceful restraint rather than overt conflict provides one possible explanation for why such female-biased variances in reproductive success in cooperative vertebrates are rarely accompanied by female-biased size dimorphisms. Intrasexual selection may nevertheless have acted more strongly on the competitive traits of males, if reproductive sharing among males is more frequently determined via overt contest competition [46].

Despite the potential importance of threats for the peaceful resolution of reproductive conflict, there remains little direct evidence to date that reproductive skew is indeed enforced by threats [9,21,38,44,80,84,85], doubtless due in part to the difficulty of testing this hypothesis. There are certainly societies in which overt interference by dominants by means of social stress [8,97,124], infanticide [8,12,95,102] or evictions [9,28,96,100] typically plays no clear role in maintaining reproductive skew [21,82,89,93,94,109,125], suggesting that any reproductive conflict has indeed been peacefully resolved. However, few if any studies of social vertebrates to our knowledge have conclusively demonstrated a role for threats of action *per se* (rather than non-punitive mechanisms) in generating such peaceful reproductive outcomes. In the many peaceful societies where subordinates never attempt to breed, or where natural instances of subordinate reproduction might conceivably have been ‘permitted’ by the dominant (e.g. due to a transient rise in *p*_crit_), the punitive actions of dominants that may routinely enforce restraint might never be observed under natural conditions. Attempts to test a role for threats by then removing the dominant may rarely provide conclusive support, as the expected reproductive upregulation among subordinates [e.g. 109,126] might also be predicted wherever non-punitive mechanisms devalue subordinate reproduction contingent on the presence of the dominant (e.g. via costs arising from competition between their litters; see above). Indeed, such offspring competition costs could also account for reproductive abstention in response to the signals of dominants [80,87,106,107,127,128], without a need to invoke a role for threats *per se*. Ultimately, the only compelling way to test a role for threats in generating reproductive skew is to experimentally force subordinates to breed at super-normal levels with a view to eliciting the predicted contingent responses of dominants (see [28,29] for just such an approach to demonstrating the role of threats in generating growth restraint in fish) or to manipulate the location of threat thresholds (e.g. by altering the cost of exercising a threat). In order to advance our understanding of the role that threats play in mediating the peaceful resolution of reproductive conflict in vertebrate societies, imaginative experiments to generate such transgressions in a biologically realistic manner while overcoming the logistical and ethical challenges entailed in doing so should now be prioritized [38,80].
